# Population reference and healthy standard blood pressure range charts in pregnancy: findings from the Born in Bradford cohort study

**DOI:** 10.1038/s41598-019-55324-2

**Published:** 2019-12-11

**Authors:** Gillian Santorelli, Debbie A. Lawlor, Jane West, Derek Tuffnell, Diane Farrar

**Affiliations:** 1 Bradford Institute for Health Research, Temple Bank House, Bradford Royal Infirmary, Duckworth Lane, Bradford, BD9 6RJ UK; 2 MRC Integrated Epidemiology Unit at the University of Bristol, Room OS11, Oakfield House, Oakfield Grove, Bristol, BS8 2BN UK; 3 Population Health Science, Bristol Medical School, University of Bristol, First Floor, Senate House, Tyndall Avenue, Bristol, BS8 1TH UK; 4 Bradford Women’s and Newborn Unit, Bradford Royal Infirmary, Bradford, BD9 6RJ UK

**Keywords:** Health care, Diseases

## Abstract

Women who develop gestational hypertension are at increased risk of adverse perinatal and longer-term outcomes. Reference charts may aid early detection of raised blood pressure (BP) and in doing so reduce adverse outcome risk. We used repeated BP measurements to produce ‘reference’ (whole population) and ‘standard’ (healthy pregnancies only) gestational-age-specific BP charts for all pregnant women (irrespective of ethnicity) and for White British (WB) and Pakistani (P) women. We included 9218 women recruited to the Born in Bradford study with 74,770 BPs. 19% of the whole population, 11% and 25% of WB and P women respectively were defined as healthy pregnancies. For reference and standard charts, for all women and each ethnic group, SBP/DBP at 12 and 20 weeks gestation was similar before rising at 37 weeks. DBP/SBP of reference charts for all women and for each ethnic group were higher than those of the corresponding standard charts. Compared to WB, P women had lower SBP/DBP at 12, 20 and 37 weeks gestation. To conclude; maternal population BP reference charts are higher compared to standard charts (healthy pregnancies) and are influenced by ethnicity.

## Introduction

Substantial cardiovascular changes occur in pregnancy to facilitate normal growth and development of the placenta and fetus. However, around 10% of women experience hypertensive disorders in pregnancy (HDP (gestational hypertension (GH) and pre-eclampsia (PE))^1,2^, and are consequently at increased risk of adverse perinatal outcomes, including caesarean and preterm birth, intrauterine growth restriction and maternal and fetal death^3–5^.

For fetal and childhood growth, there are established patterns of change. Deviation from these patterns has been used for decades to monitor healthy growth. Broadly two types of chart are used– standard and reference charts^6–8^. Standard charts aim to represent worldwide normal fetal and childhood growth. Generally, reference charts describe the growth of fetuses and children from a defined geographical area, characteristics including ethnicity (and for fetal growth, maternal characteristics including BMI) may also be considered. The appropriateness of standard versus reference charts remains contentious; the argument against reference charts (and country specific charts) is that they may normalize those at highest risk because they fail to appreciate that a woman’s socioeconomic status is a stronger predictor of health status than her genetic code and when unconstrained by nutritional and socio-economic factors, fetal and infant growth is generally consistent across populations^8,9^. Standard charts therefore describe how infants should grow and reference charts describe how infants of mothers with specific characteristics have grown.

Similarly to fetal and childhood growth, blood pressure (BP) in pregnancy is repeatedly measured and high BP first identified after 20 weeks is used to determine the diagnosis of GH and PE. Women with these conditions then have more intensive antenatal care. We and others have previously shown that BP at the start of pregnancy and change across pregnancy varies by maternal characteristics including body mass index (BMI kg/m^2^), parity^10,11^, and ethnicity^11,12^, both in normal pregnancies and in those complicated by HDP. It is therefore possible that reference charts that reflect population BP change or standard BP charts that reflect BP change in uncomplicated pregnancies might help identify women at risk earlier in pregnancy compared to diagnostic BP thresholds.

BP reference ranges for women with differing characteristics vary^13^ and the addition of BP measurements improves risk prediction models for PE and its associated adverse outcomes^14^. However, these studies were conducted in a predominantly White European population and it is clear that ethnicity can influence physiology. Compared to White Europeans, South Asians are at increased risk of insulin resistance and cardiovascular disease^15–18^. In pregnancy this is reflected in a higher incidence of gestational diabetes^19^. We have shown that compared to Pakistani (P) women, White British (WB) women start pregnancy with higher systolic blood pressure (SBP) and diastolic blood pressure (DBP), are less likely to develop GH, but are just as likely to develop PE^11^. It is likely that women who are diagnosed with GH/PE reach the diagnostic BP thresholds through different patterns of BP change (e.g. starting pregnancy, and continuing through it, with on average higher BP, having no or a less marked decline in BP in early pregnancy or a steeper rise after 20 weeks). Understanding how these differences translate into maternal BP standard and reference charts would help clarify their utility in clinical practice.

The aims of this study were to develop population reference charts (irrespective of maternal conditions, behaviours or characteristics) and standard charts (women defined as having a healthy pregnancy: no evidence of pre-existing hypertension, pre-existing diabetes or gestational diabetes, gestational hypertension or pre-eclampsia in the current pregnancy, no smoking or alcohol during pregnancy, a healthy BMI, did not deliver a preterm infant or an infant who was small for gestational age (SGA) or large for gestational age (LGA)). We stratified by ethnicity and parity.

## Methods

### Participants

**Figure 1:**
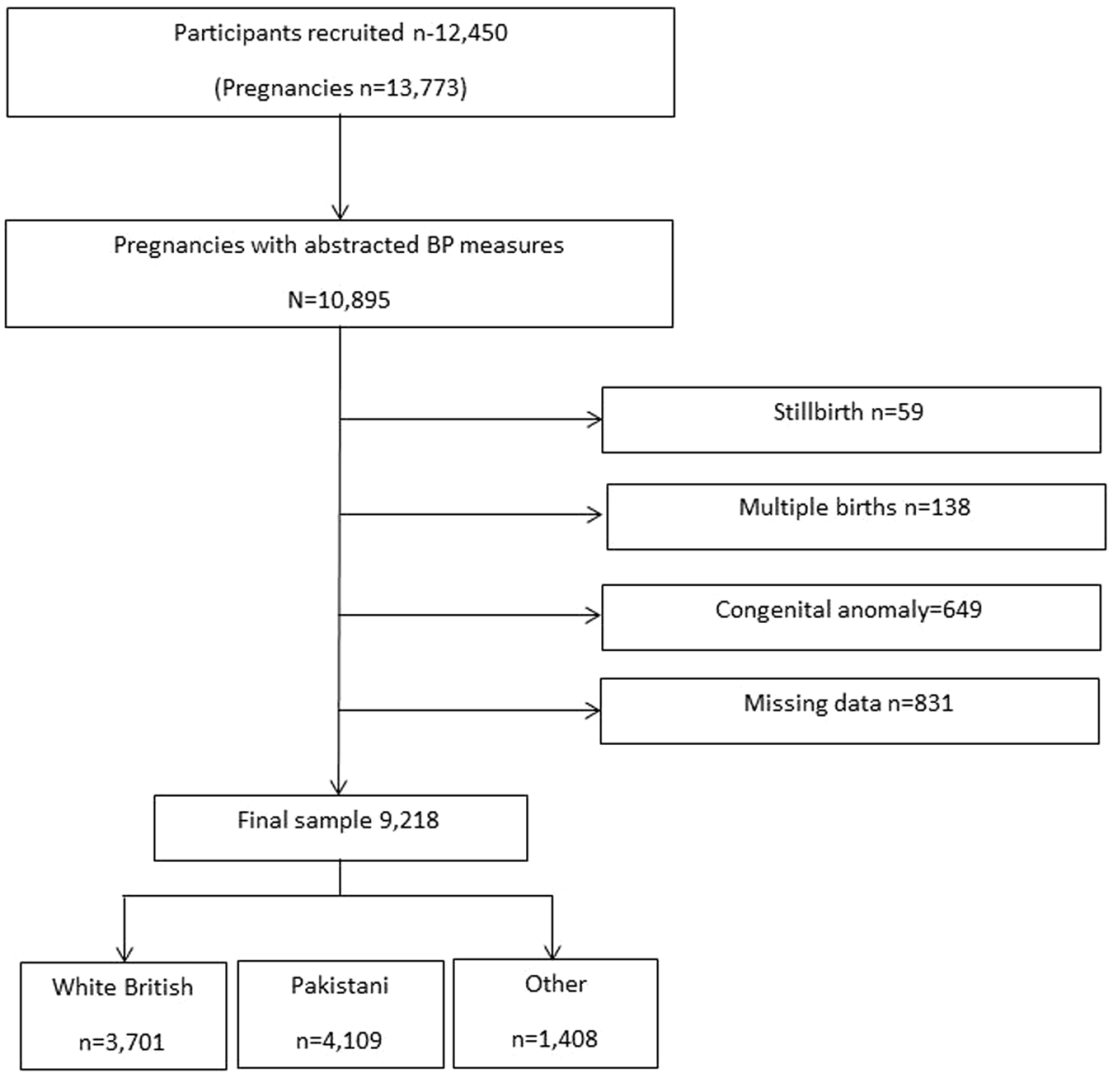
Study flow chart.

Born in Bradford (BiB) is a prospective longitudinal cohort study established to examine how genetic, nutrition, environment, behavioural and social factors impact on health and development during childhood; full details of the study methodology have been reported elsewhere^20^. At recruitment, participants gave informed written consent, or, if under 18, from a parent and/or legal guardian. Briefly, most women were recruited while attending their oral glucose tolerance test (OGTT), which is offered to all women booked for delivery at the Bradford Royal Infirmary at approximately 26–28 weeks gestation. Those who gave consent completed an interviewer-administered baseline questionnaire. The full BiB cohort recruited 12,450 pregnant women across 13,773 pregnancies between March 2007 and December 2010. The cohort for the present study included 10,895 women who had at least two BP and proteinuria measurements recorded antenatally, completed a baseline questionnaire and had birth outcomes recorded. As this is not an experimental study, protocol/analysis plan approval was not required. The following exclusions were applied: still births (n he59), multiple births (n 9)138), pregnancies resulting in the birth of children with congenital anomalies (n 38649), and women who had missing baseline questionnaire or birth outcome data (n 49831). The final cohort for this study is 9,218: 3,701 White British, 4,109 Pakistani and 1,408 women of other ethnic groups (see Fig. 1 for a flow of participants through the study).

### Maternal and neonatal measurements

BP and proteinuria measurements were recorded during pregnancy by doctors, midwives or trained health care assistants as part of routine antenatal care. Four trained research assistants abstracted this information from participant’s paper records; five percent of abstractions were checked for accuracy and error rates were less than 1%. Information on existing hypertension and existing diabetes mellitus was also obtained from the pregnancy paper records; parity and birth weight were obtained from the electronic maternity hospital records. Maternal height and weight were measured at pregnancy booking which was generally before 12 weeks gestation. Gestational age at delivery was estimated from the date of delivery and the 10 week ultrasound scan. Gender-specific birth weight centiles were calculated using the UK90 growth reference^21^. Information on congenital anomalies was obtained from the Yorkshire and Humber Congenital Anomalies Register and primary care records up to five years of age^22^.

### Definition of a healthy pregnancy

We defined a healthy pregnancy as one with no evidence of any of the following adverse pregnancy/perinatal outcomes and lifestyle characteristics: pre-existing hypertension (n = 81), pre-existing diabetes (n = 13) or developed gestational diabetes in the current pregnancy (defined using oral glucose tolerance results at 26–28 weeks gestation, using the glucose thresholds applied at the time of the study^23^; n = 718), developed gestational hypertension (defined using the thresholds: SBP ≥ 140 mm/Hg and/or DBP ≥ 90 mm/Hg on at least two occasions after 20 weeks gestation; n = 658) or pre-eclampsia (defined using the gestational hypertension thresholds with concurrent proteinuria of ≥1 + after 20 weeks gestation; n = 233), smoked (n = 1526) or consumed alcohol (n = 1859) during pregnancy, did not have a healthy BMI (defined as a BMI < 18.5 or ≥25 kg/m^2^; n = 5055), preterm delivery (defined as gestational age <37 completed weeks; n = 447), delivery of a SGA (defined as birthweight below the 10^th^ centile; n = 2351) or LGA (defined as birthweight above the 90^th^ centile; n = 373) infant.

### Statistical analysis

Multilevel models were used to produce references ranges for SBP and DBP across gestation with measurement occasion (as level 1) within women (level 2) in the whole cohort (population pregnancy reference BP ranges) and then separately in the subgroup of women defined as having a ‘healthy pregnancy’ (healthy pregnancy standard BP ranges). As a small proportion of women had many BP measurements, one measurement was randomly selected per two-week period to avoid them overly influencing the model. This was achieved by creating a random number for each record using the invnorm(uniform) command in Stata, sorting data by participant identifier, gestation period and random number, then choosing the first record within each gestation period. Models were fitted separately for SBP and DBP, with gestational age as the exposure. The shape of the BP trajectory across pregnancy was described by fitting restricted cubic splines using knot points which have been previously identified in the same dataset using linear splines (at 24, 30 and 36 weeks)^11^, with two additional outer knots at the 5^th^ and 9^th^ percentiles of the data (14 and 40 weeks)^11^. The median time of the first antenatal BP measurement (12 weeks) was used as the baseline gestational age and was included as a level 1 random effect.

Reference (irrespective of health conditions, behaviors or characteristics) and standard (healthy pregnancies) BP range charts were derived for all women (irrespective of ethnicity) and separately for the two largest ethnic groups (White British (WB) and Pakistani (P) women). There were too few women of other ethnicities to conduct meaningful analysis. MLwiN version 2.32 was used to fit the multilevel models through Stata^24^. Stata version 13.1 (Stata Corp, College Station, Texas, USA) was used for all other analyses.

### Ethics approval

Ethics approval for the study was granted by Bradford National Health Service Research Ethics Committee (07/H1302/112), all research was performed in accordance with relevant guidelines and regulations.

## Results

The characteristics of all women included in the sample and stratified by WB and P ethnic group are presented in Table 1. There were 74,770 BP measurements taken between 9 and 41 weeks gestation, with a median of 8 measurements per women (IQR 7 to 9). Overall, 19% of women were defined as having a healthy pregnancy, with fewer WB women having healthy pregnancies compared to P women (11.5% vs 25.2%).

**Table 1:** Maternal characteristics, overall and by ethnic group.

	All (n = 9218)^c^		White British (n = 3701)		Pakistani (n = 4109)	
	N	Median (IQR) or %	N	Median (IQR) or %	N	Median (IQR) or %
Number of measures^a^	74,770	8 (7, 9)	30,826	9 (8, 10)	32,700	8 (7, 9)
**Parity**						
Nulliparous	3004	40.0	1727	48.2	1277	32.5
Multiparous	4506	60.0	1852	51.8	2654	67.5
Pre-existing hypertension	81	0.9	45	1.2	28	0.7
Pre-existing diabetes (type 1 & 2)	13	0.1	6	0.2	6	0.2
Gestational hypertension	658	7.1	397	10.7	181	4.4
Pre-eclampsia	233	2.5	102	2.8	96	2.3
Gestational diabetes	718	7.8	170	4.6	432	10.5
Preterm delivery	447	4.9	183	4.9	187	4.6
Large for gestational age	373	4.1	232	6.3	98	2.4
Small for gestational age	2351	25.5	682	18.4	1293	31.5
Alcohol during pregnancy	1859	20.2	1640	44.3	17	0.4
Smoked during pregnancy	1526	16.6	1238	33.5	142	3.5
BMI < 18.5 or ≥25.0	5055	54.8	2099	56.7	2256	54.9
Healthy pregnancy^b^	1790	19.4	426	11.5	1,036	25.2

^a^Number of blood pressure measurements per woman following random selection of one measure per two week period of pregnancy.

^b^Healthy pregnancies are defined as those without medical conditions (has pre-existing hypertension, has existing diabetes or developed gestational diabetes, developed pre-eclampsia) and adverse pregnancy outcomes (pre-term birth, small for gestational age or lagre for gestational age infant) and unhealthy lifestyle characteristics (smoking and drinking alcohol during pregnancy, unhealthy BMI).

^c^Composed of the following ethnic groups: White British = 3701; Pakistani = 4109; Other ethnicity = 1408.

**Figure 2:**
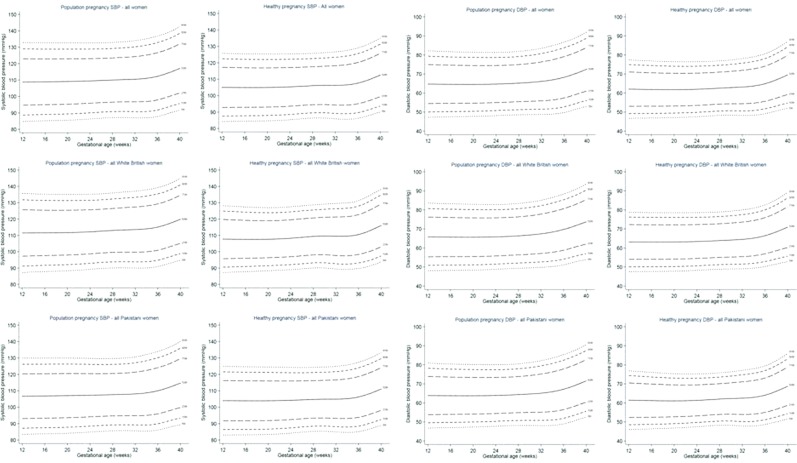
a and b: Reference (population) and standard (healthy pregnancies) blood pressure charts for SBP and DBP between 12 and 40 weeks gestation for all (irrespective of ethnicity), White British and Pakistani women.

The reference and standard charts for SBP and DBP between 12 and 40 weeks gestation are shown for all women and for WB and P women in Fig. 2a,b, and by parity in Supplementary Figs. 1 and 2. The predicted mean SBP and DBP with 95% CIs at 12, 20 and 37 weeks are presented overall and stratified by ethnicity in Table 2 and further stratified by parity in Supplementary Table 1.

**Table 2:** Mean (95% confidence intervals) systolic blood pressure and diastolic blood pressure at gestational age of 12, 20 and 37 weeks for standard and reference charts, stratified by ethnicity.

	Reference charts – All pregnancies			Standard charts - Healthy pregnancies		
	12 weeks	20 weeks	37 weeks	12 weeks	20 weeks	37 weeks
**All women**						
SBP	108.8 (84.7, 132.9)	109.1 (85.5, 132.7)	112.5 (88.1, 136.9)	105.0 (84.2, 125.8)	104.9 (84.6, 125.3)	107.7 (86.0, 129.4)
DBP	64.7 (47.2, 82.2)	64.5 (47.7, 81.4)	68.2 (49.9, 86.4)	62.1 (46.7, 77.4)	61.8 (47.1, 76.4)	64.8 (49.2 80.5)
**White British women**						
SBP	111.5 (87.2, 135.7)	111.7 (88.3, 135.1)	115.2 (91.1, 139.3)	107.7 (87.2, 128.2)	107.6 (88.3, 126.9)	110.6 (89.7, 131.5)
DBP	65.8 (48.0, 83.6)	65.6 (48.3, 83.0)	69.5 (51.1, 87.9)	63.1 (47.6, 78.7)	63.1 (47.8, 78.5)	66.5 (50.4, 82.6)
**Pakistani women**						
SBP	106.7 (83.4, 129.9)	107.0 (84.1, 130.0)	110.3 (86.5, 134.2)	104.0 (83.0, 124.9)	103.8 (83.3, 124.5)	106.4 (84.8, 128.0)
DBP	63.9 (46.8, 81.0)	63.7 (47.3, 80.2)	67.1 (49.2, 85.1)	61.5 (46.0, 76.9)	61.0 (46.8, 75.3)	64.0 (48.9, 79.0)

### Reference vs standard charts

For both reference and standard charts and for all women, WB and P women, SBP and DBP at 12 and 20 weeks gestation was similar before rising by ~2 mm/Hg to 3 mm/Hg at 37 weeks (see Fig. 2a,b). The DBP and SBP predicted means of the reference charts for all women (irrespective of ethnicity), WB and P women, were higher than those of the corresponding standard charts. The 95% CIs of each group’s reference charts were between 3 mm/Hg and 8 mm/Hg wider than each group’s equivalent standard chart.

### Ethnic differences: reference charts

Compared to WB women, P women had lower mean SBP and DBP (~5 mm/Hg and ~2–3 mm/Hg respectively) at each of the three gestational ages (12, 20 and 37 weeks). For both ethnic groups mean SBP at 12 and 20 weeks and DBP at 12 and 20 weeks was similar before rising at 37 weeks gestation by ~3–4 mm/Hg (see Table 2 and Fig. 2a,b).

### Ethnic differences: standard charts

Similar to reference charts, the standard charts of P women had lower mean SBP and DBP across pregnancy compared to WB women. Within each ethnic group, mean SBP at 12 and 20 weeks and DBP at 12 and 20 weeks, was similar before rising at 37 weeks gestation by ~3–4 mm/Hg (see Table 2 and Fig. 2a,b).

### Parity differences: standard versus reference charts

The SBP and DBP predicted means of the standard charts for all women, WB and P women were lower than those of the corresponding reference charts. The upper CIs were higher and lower CIs lower for standard charts compared to the reference charts.

### Parity differences: nulliparous versus multiparous charts

For both standard and reference charts, the predicted mean SBP for all (irrespective of ethnicity) and WB nulliparous women were generally similar to multiparous women at 12 weeks and 20 weeks gestation, but higher by ~2 mmHg at 37 weeks of pregnancy. The standard charts of P nulliparous compared to P multiparous women had slightly lower mean SBP at 12 and 20 weeks, but higher SBP at 37 weeks. The predicted mean DBP for all and WB nulliparous women were generally similar to the corresponding charts of multiparous women at each time point. The reference charts of P nulliparous compared to multiparous women had lower predicted mean DBP at 12 and 20 weeks and higher mean DBP at 37 weeks. For standard charts, P nulliparous and multiparous predicted DBP was similar at 12 and 20, at 37 weeks however the mean DBP of nulliparous women exceeded that of multiparous women (See Sup. Table 1 and Sup. Fig 1 and 2).

## Discussion

Using data from a large prospective birth cohort, we have produced population reference (for all pregnancies irrespective of maternal medical conditions, behaviours or characteristics) and standard (for normally defined pregnancies) BP charts and examined the influence of ethnicity and parity on these charts. We found that BP range varies as gestation progresses, that reference compared to standard charts have higher BP ranges across pregnancy and that the reference and standard charts of P compared to the corresponding charts of WB women are lower throughout pregnancy. The SBP ranges of charts for multiparous compared to corresponding charts of nulliparous women were similar, whereas the DBP ranges of charts were generally lower for multiparous compared to the corresponding charts of nulliparous women.

Reference or standard BP charts for pregnant women are not currently recommended for clinical practice, BP thresholds are used to define high BP and in the diagnosis of HDP^25–27^. Placental insufficiency underlies the increased risk of growth restriction and preterm birth associated with HDP, however placental insufficiency may be present for some time prior to its identification or to the diagnosis of a HDP. It is possible that the degree of BP increase and pattern of BP change as well as BP level reached may contribute to the occurrence of placental insufficiency and subsequent development of adverse outcomes^28,29^. Therefore further research is needed to explore the use of BP reference and standard BP charts in the identification of deviations from usual BP change patterns, which may help in the earlier identification of women at increased risk of HDP associated adverse outcomes.

We have previously shown that the BP trajectories of P women vary from those of WB women: they are lower throughout the first half of pregnancy before increasing in the second half to equal the BP level of WB women^11^. Using current BP threshold criteria to define HDP^27^ these differences produce a lower prevalence of GH, but similar prevalence of PE for P compared to WB women^11^. We have shown that when all P pregnancies and when only ‘normal’ pregnancies are included, the reference ranges for P women are lower across pregnancy compared to WB women suggesting that deviation from the normal range will occur at a lower BP level for P compared to WB women. The lower BP levels of the P women’s trajectories compared to WB women, means that they would need greater increases in BP to achieve the current thresholds that define a HDP and these greater increases may contribute to a higher risk of adverse outcomes (compared to WB women).

Concerns regarding the use of BP reference (that describe population norms for all pregnancies) and standard (for healthy pregnancies only) charts are similar to those surrounding the use of fetal and infant growth reference charts (that describe how infants in a particular population have grown) compared to standard charts (that describe how unrestricted growth should occur). Reference BP charts may better describe how BP changes across the whole population, but these charts may underestimate risk in women most vulnerable, especially in a high risk population where average BP ranges may be higher than for the average (minority) ‘healthy’ (without identified risk characteristics) pregnancy. Standard BP charts that describe normal pregnancy BP ranges may over or under identify risk if BP change pattern deviations are not good predictors of adverse perinatal outcomes and we have shown that ethnicity can influence BP range levels.

## Supplementary information

Supplementary Information

## Data Availability

The Born in Bradford Study allows bone fide researchers to access data. Full details of how to do this are provided on the study web pages (https://borninbradford.nhs.uk/research/how-to-access-data/).
