# Publisher Correction: Population reference and healthy standard blood pressure range charts in pregnancy: findings from the Born in Bradford cohort study

**DOI:** 10.1038/s41598-020-61787-5

**Published:** 2020-03-11

**Authors:** Gillian Santorelli, Debbie A. Lawlor, Jane West, Derek Tuffnell, Diane Farrar

**Affiliations:** 1 Bradford Institute for Health Research, Temple Bank House, Bradford Royal Infirmary, Duckworth Lane, Bradford, BD9 6RJ UK; 2 MRC Integrated Epidemiology Unit at the University of Bristol, Room OS11, Oakfield House, Oakfield Grove, Bristol, BS8 2BN UK; 3 Population Health Science, Bristol Medical School, University of Bristol, First Floor, Senate House, Tyndall Avenue, Bristol, BS8 1TH UK; 4 Bradford Women’s and Newborn Unit, Bradford Royal Infirmary, Bradford, BD9 6RJ UK

Correction to: *Scientific Reports* 10.1038/s41598-019-55324-2, published online 11 December 2019

This Article contains a typographical error in the Methods section under subheading ‘Participants’ where,

“The following exclusions were applied: still births (n he59), multiple births (n 9)138), pregnancies resulting in the birth of children with congenital anomalies (n 38649), and women who had missing baseline questionnaire or birth outcome data (n 49831).”

should read:

“The following exclusions were applied: still births (n = 59), multiple births (n = 138), pregnancies resulting in the birth of children with congenital anomalies (n = 649), and women who had missing baseline questionnaire or birth outcome data (n = 831).”

In addition there is a typographical error in the Methods section under subheading ‘Statistical Analysis’ where,

“The shape of the BP trajectory across pregnancy was described by fitting restricted cubic splines using knot points which have been previously identified in the same dataset using linear splines (at 24, 30 and 36 weeks), with two additional outer knots at the 5th and 9th percentiles of the data (14 and 40 weeks).”

should read:

“The shape of the BP trajectory across pregnancy was described by fitting restricted cubic splines using knot points which have been previously identified in the same dataset using linear splines (at 24, 30 and 36 weeks), with two additional outer knots at the 5th and 95th percentiles of the data (14 and 40 weeks).”

