# Supplementary material for: Population reference and healthy standard blood pressure range charts in pregnancy: findings from the Born in Bradford cohort study
Source: Sci Rep. 2019 Dec 11;9:18847. doi: 10.1038/s41598-019-55324-2 (PMC6906473; doi:10.1038/s41598-019-55324-2)
Supplement: Supplementary file 1 — Supplementary Information [file 41598_2019_55324_MOESM1_ESM.pdf]

Population reference and healthy standard blood pressure range charts in pregnancy: findings from the Born in Bradford cohort study

Gillian Santorelli, Debbie A Lawlor, Jane West, Derek Tuffnell, Diane Farrar

Supplementary table 1: Mean (95% reference range) systolic blood pressure (SBP) and diastolic blood pressure (DBP) at gestational age of 12, 20 and 37 for standard and reference charts, stratified by ethnicity and parity

	Reference charts -All pregnancies			Standard charts -Healthy pregnancies		
	**12 weeks**	**20 weeks**	**37 weeks**	**12 weeks**	**20 weeks**	**37 weeks**
**All women**						
**SBP**						
Nulliparous	108.7 (84.8, 132.6)	109.3 (85.6, 132.9)	113.4 (88.7, 138.1)	105.5 (85.0, 126.0)	105.5 (85.7, 125.4)	108.8 (87.2, 130.4)
Multiparous	108.8 (84.7, 133.0)	108.9 (85.5, 132.3)	111.8 (87.7, 135.9)	104.6 (83.6, 125.6)	104.4 (83.8, 125.0)	106.8 (85.2, 128.4)
**DBP**						
Nulliparous	64.2 (46.8, 81.6)	64.2 (47.5, 81.0)	68.7 (50.3, 87.2)	62.0 (46.9, 77.2)	61.4 (47.0, 76.0)	65.9 (49.8, 81.9)
Multiparous	65.0 (47.5, 82.5)	64.7 (47.8, 81.6)	67.7 (49.7, 85.8)	61.4 (46.3, 76.6)	61.3 (47.2, 75.4)	64.0 (48.8, 79.2)
**White British women**						
**SBP**						
Nulliparous	111.2 (87.1, 135.3)	111.7 (88.0, 135.4)	115.6 (91.1, 140.1)	107.8 (86.2, 129.3)	108.4 (87.2, 129.6)	112.0 (92.0, 132.0)
Multiparous	111.7 (87.4, 135.9)	111.6 (88.6, 134.5)	114.8 (91.0, 138.6)	107.0 (87.5, 126.5)	107.8 (88.1, 127.4)	110.9 (90.4, 131.3)
**DBP**						
Nulliparous	65.3 (47.4, 83.1)	65.4 (48.0, 82.7)	69.9 (51.5, 88.3)	63.1 (46.3, 78.0)	63.0 (48.0, 78.0)	67.2 (52.1, 82.3)
Multiparous	66.3 (48.5, 84.1)	65.8 (48.5, 83.2)	69.1 (50.8, 87.4)	62.9 (48.0, 77.9)	63.4 (48.4, 78.5)	66.6 (51.2, 82.1)
**Pakistani women**						
**SBP**						
Nulliparous	105.7 (83.2, 128.1)	106.4 (84.0, 128.7)	110.8 (86.5, 135.1)	104.1 (83.7, 124.5)	104.3 (84.1, 124.5)	107.4 (84.9, 130.0)
Multiparous	107.0 (83.5, 130.6)	107.3 (84.1, 130.4)	110.1 (86.6, 133.6)	103.8 (82.6, 125.0)	103.5 (82.9, 124.0)	105.7 (84.9, 126.4)
**DBP**						
Nulliparous	62.9 (46.4, 79.3)	63.1 (47.0, 79.1)	67.6 (49.0, 86.3)	61.5 (46.3, 76.6)	61.5 (46.8, 76.3)	65.1 (49.0, 81.3)
Multiparous	64.3 (47.0, 81.5)	64.0 (47.4, 80.5)	66.9 (49.3, 84.5)	61.5 (46.0, 77.0)	60.8 (46.8, 74.8)	63.2 (48.9, 77.5)

Supplementary figure 1: Reference (population) and standard (healthy pregnancies) charts with centiles for SBP and DBP between 12 and 40 weeks gestation for all women (irrespective of ethnicity), White British and Pakistani nulliparous women

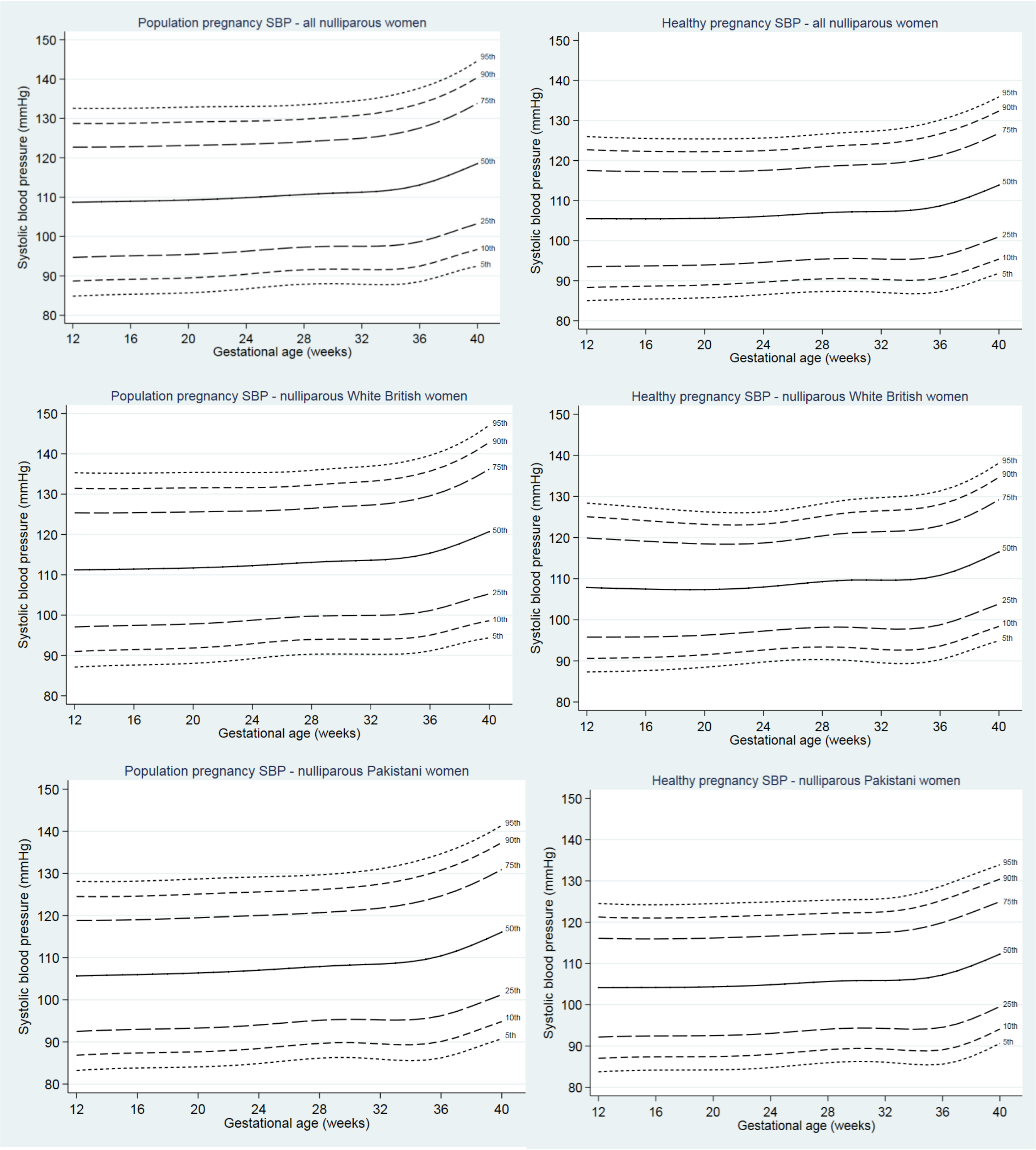

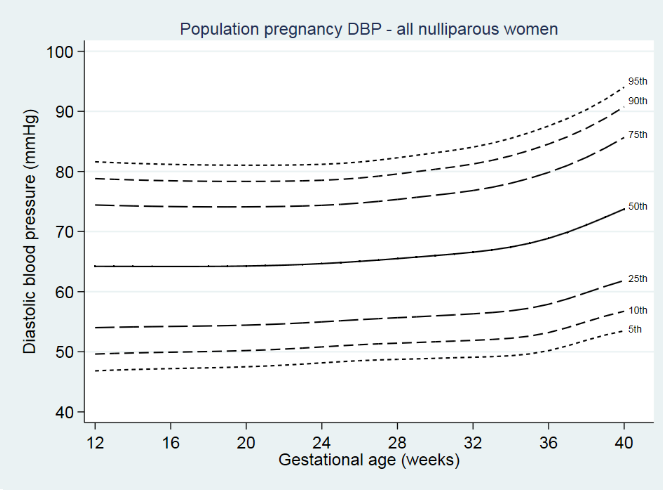
Population pregnancy DBP - all nulliparous women
Diastolic blood pressure (mmHg)
100
90
80
70
60
50
40
95th
90th
75th
50th
25th
10th
5th
12
16
20
24
28
32
36
40
Gestational age (weeks)

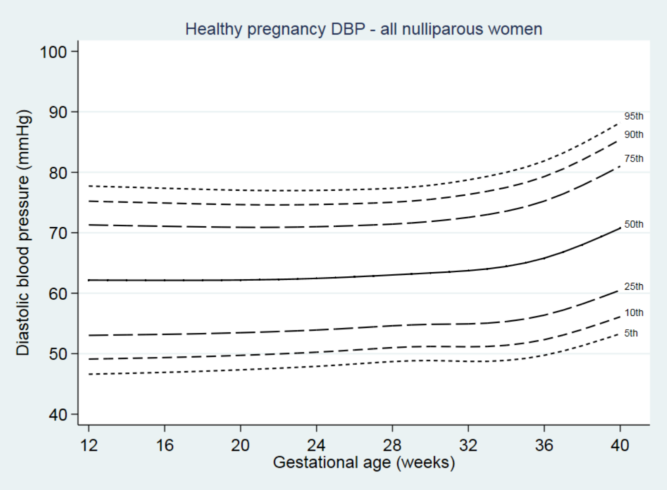
Healthy pregnancy DBP - all nulliparous women
Diastolic blood pressure (mmHg)
100
90
80
70
60
50
40
95th
90th
75th
50th
25th
10th
5th
12
16
20
24
28
32
36
40
Gestational age (weeks)

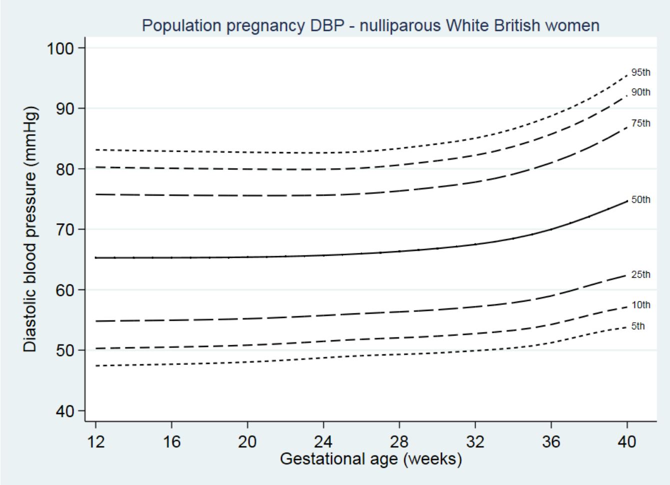
Population pregnancy DBP - nulliparous White British women
Diastolic blood pressure (mmHg)
100
90
80
70
60
50
40
95th
90th
75th
50th
25th
10th
5th
12
16
20
24
28
32
36
40
Gestational age (weeks)

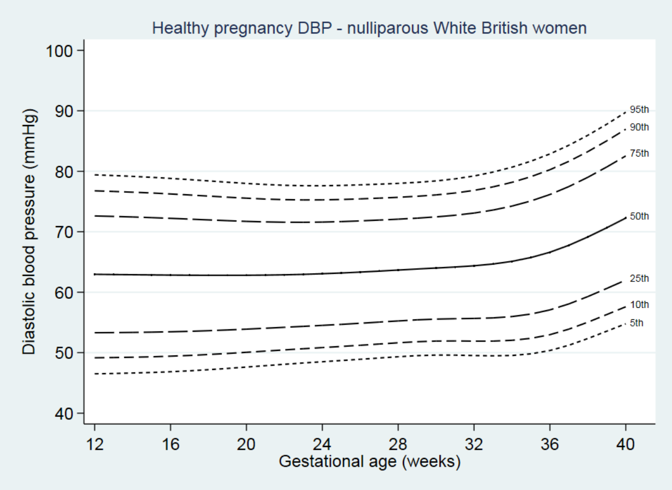
Healthy pregnancy DBP - nulliparous White British women
Diastolic blood pressure (mmHg)
100
90
80
70
60
50
40
95th
90th
75th
50th
25th
10th
5th
12
16
20
24
28
32
36
40
Gestational age (weeks)

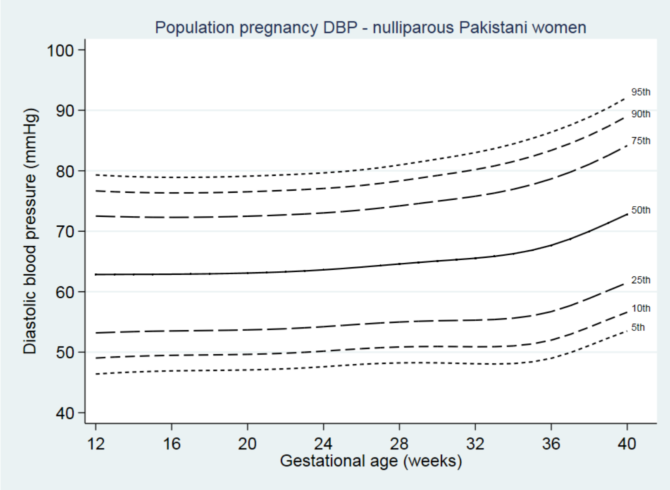
Population pregnancy DBP - nulliparous Pakistani women
Diastolic blood pressure (mmHg)
100
90
80
70
60
50
40
95th
90th
75th
50th
25th
10th
5th
12
16
20
24
28
32
36
40
Gestational age (weeks)

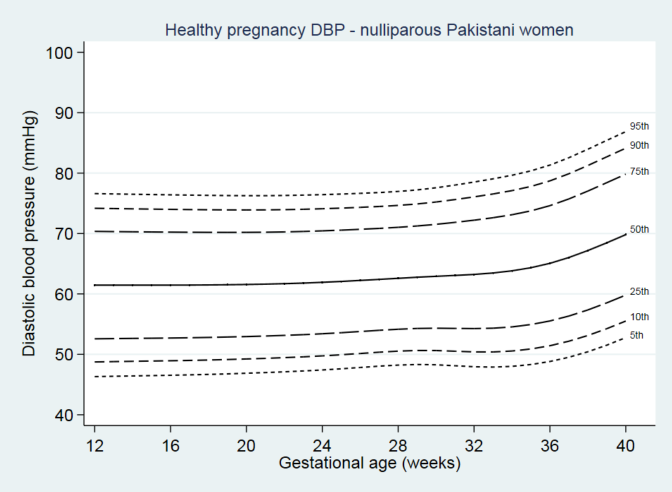
Healthy pregnancy DBP - nulliparous Pakistani women
Diastolic blood pressure (mmHg)
100
90
80
70
60
50
40
95th
90th
75th
50th
25th
10th
5th
12
16
20
24
28
32
36
40
Gestational age (weeks)

Supplementary figure 2: Reference (population) and standard (healthy pregnancies) charts with centiles for SBP and DBP between 12 and 40 weeks gestation for all women (irrespective of ethnicity), White British and Pakistani multiparous women

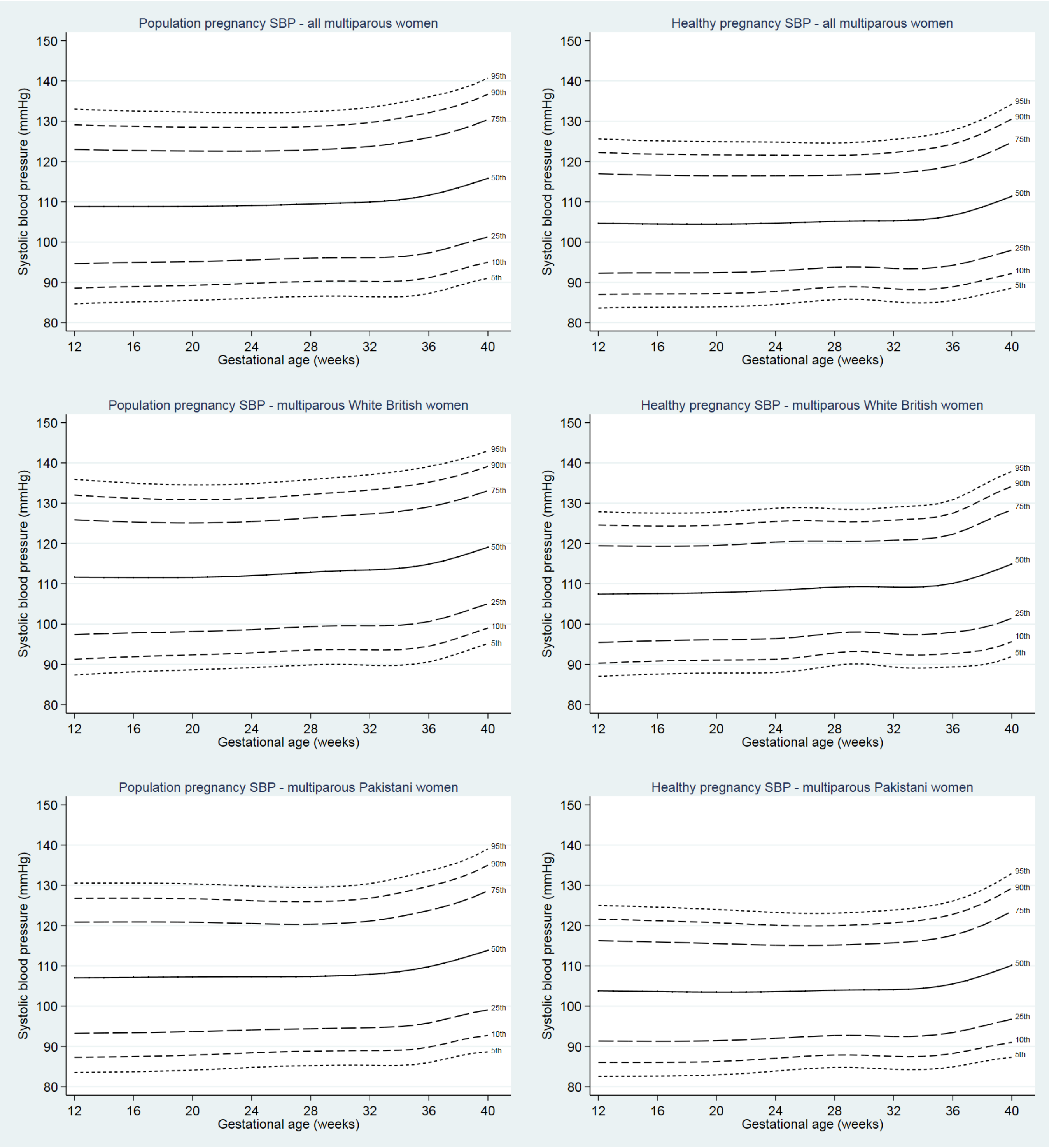

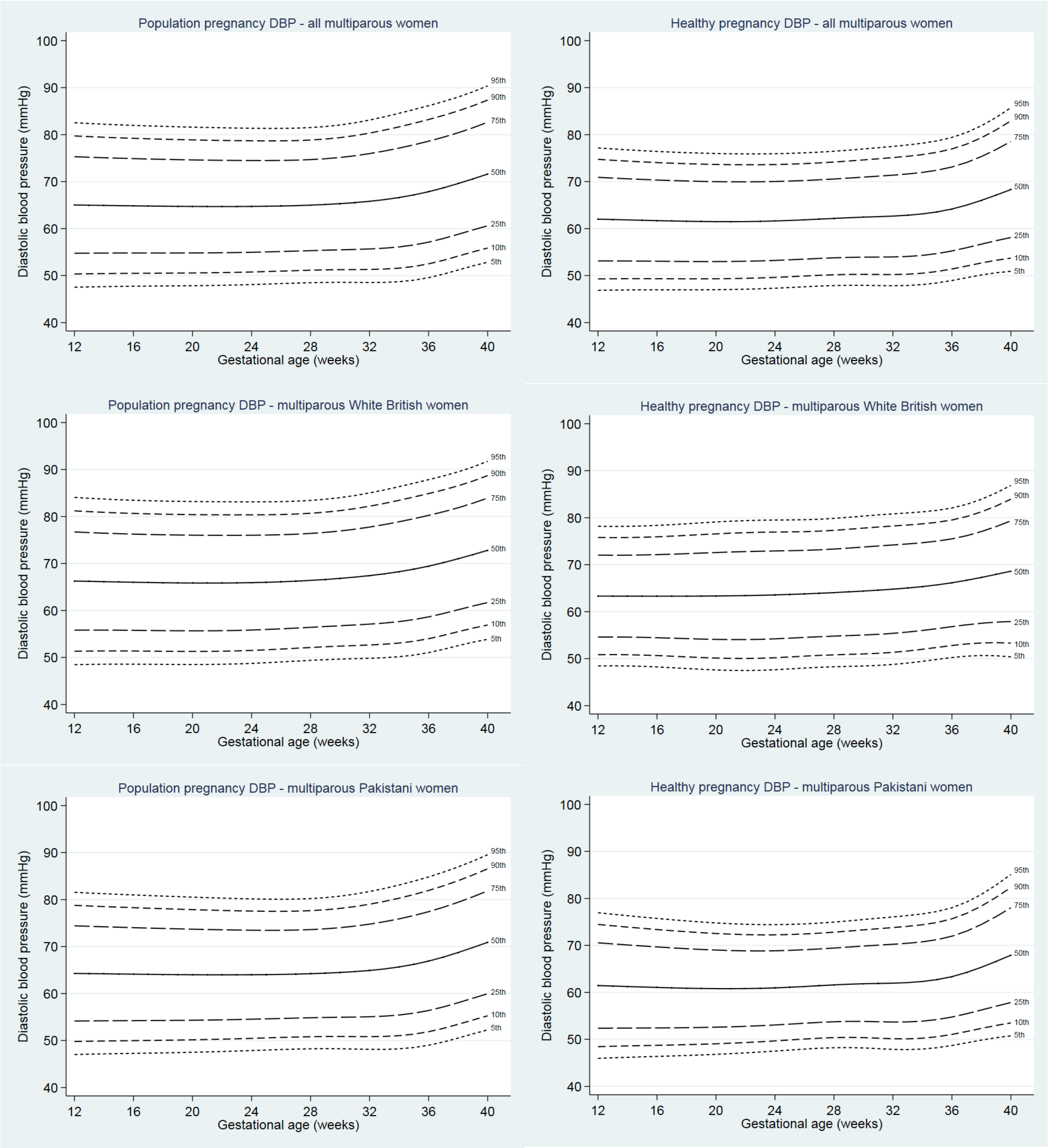
Population pregnancy DBP - all multiparous women
Healthy pregnancy DBP - all multiparous women
Population pregnancy DBP - multiparous White British women
Healthy pregnancy DBP - multiparous White British women
Population pregnancy DBP - multiparous Pakistani women
Healthy pregnancy DBP - multiparous Pakistani women
Diastolic blood pressure (mmHg)
Gestational age (weeks)
40
50
60
70
80
90
100
12
16
20
24
28
32
36
40
95th
90th
75th
50th
25th
10th
5th
